# Best practice models of aged-care implemented for First Nations people: a systematic review aligned with the Good Spirit Good Life quality of life principles

**DOI:** 10.1186/s12877-024-04781-0

**Published:** 2024-02-29

**Authors:** Caleb Rivers, Emma Haynes, Dina LoGiudice, Kate Smith, Dawn Bessarab

**Affiliations:** 1 Good Spirit Good Life Centre of Research Excellence, Centre for Aboriginal Medical and Dental Health, Medical School, University of Western Australia, Perth, Australia; 2 Centre for Aboriginal Medical and Dental Health, Medical School, University of Western Australia, Perth, Australia; 3 Department of Medicine, University of Melbourne, Melbourne, Australia; 4 Department of Aged Care, Royal Melbourne Hospital, Melbourne, Australia; 5 School of Medicine, M303, Crawley, WA 6009 Australia

**Keywords:** First Nations people, Older, Models of care, Quality of life, Systematic review

## Abstract

**Background:**

Aged-care programs that are based in First Nations worldviews are believed to translate to improved quality of life for First Nations Elders. First Nations perspectives of health and well-being incorporates social and cultural determinants in addition to traditional Western biomedical approaches. This is exemplified by the Good Spirit Good Life (GSGL) framework, which comprises 12 strength-based factors determined by First Nations Elders as constituting culturally appropriate ageing. Our objective was to conduct a systematic review of existing aged care models of practice to determine the degree of alignment with the GSGL framework. Recommendations of the national Australian Royal Commission into Aged Care Quality and Safety informed this work.

**Methods:**

We conducted a systematic search of academic and grey literature in the PubMed, Scopus, Ovid Embase, and Informit online databases. Inclusion criteria comprised English language, original research describing the implementation of First Nations culturally appropriate aged care models, published before August 2022. Research that was not focused on First Nations Elders’ perspectives or quality of life was excluded. We subsequently identified, systematically assessed, and thematically analyzed 16 articles. We assessed the quality of included articles using the Aboriginal and Torres Strait Islander Quality Assessment Tool (ATSIQAT), and the Joanna Briggs Institute (JBI) critical appraisal tool for qualitative research.

**Results:**

Most studies were of medium to high quality, while demonstrating strong alignment with the 12 GSGL factors. Nine of the included studies detailed whole service Models of care while 7 studies described a single program or service element. Thematic analysis of included studies yielded 9 enablers and barriers to implementing models of care.

**Conclusions:**

Best-practice First Nations aged care requires a decolonizing approach. Programs with strong adherence to the 12 GSGL factors are likely to improve Elders’ quality of life.

**Supplementary Information:**

The online version contains supplementary material available at 10.1186/s12877-024-04781-0.

## Introduction

Aboriginal and Torres Strait Islander people are the two distinct, broadly categorized and recognized cultural groups that comprise the Indigenous population in Australia. Collectively referred to as First Nations people in this paper, these groups historically occupied the mainland continent and surrounding islands of present-day Australia. Though constituting approximately 3.8% of the Australian population [1, 2], First Nations Australians experience a greater burden of adverse health outcomes compared to their non-Indigenous counterparts [3]. Colonization has adversely impacted the health of First Nations populations globally, with systematic means including lack of appropriate service provision perpetuating this legacy [3].

Health and wellbeing inequalities that burden First Nations populations are compounded by a lack of culturally appropriate health and aged care services. A largely Western medical approach may fail to consider that family, kinship, community and country are fundamental determinants to First Nations health and wellbeing [4]. Implementing systemic delivery of culturally-appropriate health and aged care services has the potential to address social determinants of health [4], increasing quality of life and life expectancy [3].

The Royal Commission into Aged Care Quality and Safety highlights the urgency around planning for culturally safe care and accessibility, to meet the needs of the projected number of older First Nations people in Australia [5, 6]. The life expectancy for First Nations people from Australia, Canada, USA and New Zealand is increasing [7]. The older (45-plus) population in Australia is projected to triple by 2050, with the highest growth rate expected for the oldest age groups over 85 years [8]. Many of the ageing First Nations demographic in Australia today are Stolen Generation survivors.

Colonization in Australia, as in other parts of the world marked a period of policy enactment for the purpose of control, disempowerment, and exploitation of First Nations populations. The Stolen Generation in Australia serves as a contemporary reference point to this legacy, constituting the removal of Indigenous children from their parents and families by authorities during the nineteenth and twentieth centuries. The profound effects that this exerted on the quality of life of Indigenous populations continues to have ramifications today [9].

Addressing culturally safe care and accessibility for First Nations people in Australia is especially urgent as Stolen Generations members will be eligible for aged care services as of 2023 [5, 6, 10], given conventional aged care approaches may not adequately address medical and social needs experienced by this group [10]. For example, entering into care facilities may trigger childhood trauma from earlier institutionalization [11].

Recognizing the need to improve aged care for First Nations people, the Centre for Aboriginal Medical and Dental Health at the University of Western Australia co-designed the Good Spirit Good Life (GSGL) Tool and Framework for older First Nations people. The purpose of the GSGL tool as a culturally appropriate, strength-based instrument, is to measure and provide strategies for the QoL of older First Nations Australians [12]. The tool is a response to an identified gap whereby conventional assessment tools do not accurately capture, value, or prioritize the lived experiences of First Nations Australian Elders. The GSGL tool provides a framework for supporting Elders to have a good life and a strong inner spirit; including appropriate assessment and recommendations that will inform quality, culturally meaningful health, and aged care for First Nations Elders [13]. The GSGL framework comprises 12 interconnected QoL factors, including connection to family and friends, community, country, culture, health, Elder role, respect, spirituality, supports and services, safety and security, future planning and basic needs [13]. The GSGL tool was designed and validated with older First Nations Australians from urban and regional areas in Western Australia and Victoria [12].

Although First Nations Elders have identified factors that are important to wellbeing/QoL [4, 14–28], the GSGL tool is the first study to develop a practical QoL assessment that has informed health and aged care specifically for older First Nations people. Thus, concurrent with the review reported here, the GSGL tool has been trialed as a supplementary assessment for First Nations peoples by the Australian Department of Health and Aged Care in the National single aged care assessment (2023) and is widely available for use by health and aged care services to inform care for First Nations Elders, accessible from http://www.iawr.com.au/gsgl. As described further below, although the implementation of domains of the GSGL informs our analysis of the reviewed publications, there is still a need for greater/broader implementation into policy and practice of other successful research outcomes with First Nations people, to reduce health inequity [29].

We report here on a systematic review conducted to inform the broader implementation of the GSGL framework for First Nations aged care into best practice service delivery models. This systematic review investigated the implementation of First Nations best practice models of aged care globally, to determine underlying principles and practices, enablers and challenges, and identify common themes and experiences amongst both Elder First Nation’s people, their carers, and service providers in both residential and community care.

## Materials and methods

This systematic review was undertaken by the Good Spirit, Good Life Centre for Research Excellence in First Nations Ageing at the University of Western Australia, following the PRISMA guidelines for systematic reviews [30]. This systematic review was not pre-registered with PROSPERO. A protocol was not prepared for this systematic review.

### Self-location

The interrogation of positionality is critical in establishing the rigor and trustworthiness of qualitative research [31] as it reveals how authors’ positions, assumptions and knowledge inform the research processes. When authors identify their lived experience and connection to Indigenous communities, it can demonstrate cultural humility and provide insight to the lens in which the research is being undertaken. Locating who the authors are, in relation to the context that they are writing, the authors are acknowledging potential biases, limitations and providing accountability mechanisms [32].

DB is from the Bard and Yjindabandi people in Western Australia and is a senior Indigenous researcher with expertise in qualitative and Indigenous research methodologies. She is a social worker with over 30 years of extensive experience and knowledge of cultural ways of working and applying a critical lens to all stages of the research process. CR has Gooniyandi and Kija heritage from Halls Creek on his fathers’ side of the family, and Wongi and Yamaji from Wiluna on his mothers’ side. CR completed a Bachelor of Biomedical Science at the University of Western Australia in 2018. EH is a non-Indigenous ally with qualitative research expertise; her previous research collaborations guided by DB’s application of a critical lens has informed the process of paying attention to relationships, accepting philosophical discomfort, and thinking critically about power dynamics, which all contribute to transformative writing. DL is a non-Indigenous aged care physician, who has collaborated with First Nations academics and researchers for over 20 years, focusing on the needs of older First Nations people living within their communities. These collaborations including with DB, have led to ongoing reflective thinking, and understanding about approaches to First Nations research and ways to privilege First Nations voices, with respect, genuine intent, and critical allyship. DL continues to work with First Nations Elders and their carers within the health services sector. KS is a non-Indigenous ally and occupational therapist with over 20 years expertise in older First Nations health and wellbeing research. KS was initially based as a clinical researcher in the remote town of Derby and surrounding communities, where guiding Elders, colleagues and community members first informed her cultural practice and lens, further developed in Perth. Indigenous research methodologies are central to her collaborative work including the co-design of the Lungurra Ngoora partnership model of care and Good Spirit Good Life quality of life package. All authors had concurrent experiences of taking care of older family members or caring for older patients. Central to the reiterative process of undertaking the review was the collaboration between CR and experienced researchers DB, DL and EH. Data analysis included a triangulation of review processes where first pass analyses were conducted by CR and EH, and then any anomalies were discussed with DL and DB. Similar processes occurred with determining inclusions and exclusions, assessments using quality measures and alignment with the GSGL factors and collecting data from reports. CR performed the literature search and applied inclusion and exclusion criteria with guidance from EH. CR and EH did the initial thematic analysis, DL and DB contributed to the reiterative refinement of themes, and all authors collaboratively explored and concurred on the findings.

### Systematic database literature search of academic and grey literature

Our research topic was broken down into the key terms “First Nations people”, “older”, “models of care”, and “quality of life” to develop the database search strings using appropriate and relevant synonyms. The full list of search strings is detailed in Supplementary Table 1. We conducted a systematic search of academic and grey literature in the PubMed (last searched 18/7/22), Scopus (last searched (13/7/22), Ovid Embase (last searched (last searched 13/7/22) and Informit online databases (last searched 7/6/22) [33]. The database searches were supplemented by hand searching.

Using the Pubmed version of the search string as the standard, we entered these into the Bond University Polyglot Search Translator to transcribe the search string into the appropriate version, including syntax and Boolean operators for each of the other 3 databases [34]. The title/ abstract filter was used for each of the databases.

### Inclusion/Exclusion process of publications identified from the literature search

Publications were included in this review if they were English-language original research, describing evidence relating to the implementation of First Nations culturally appropriate aged care models grounded in community-identified quality of life factors (either whole of service delivery or single program only), and if they focused on strategies to improve quality of life based on patient, family, and carer satisfaction. Following this primary inclusion process, publication focus criteria further established inclusion determinants related to ageing well; including attitudes, concepts, cultural aspects, definitions, associated terms, and implementations of principles translated into practice. Publication types include original research including qualitative, quantitative or mixed methods; grey literature; information obtained from government publications, peak bodies or organisational reports; website information, and if full text available. Exclusion of publications directly employed publication focus and type criteria. Types of publications excluded included systematic reviews, literature reviews (including included articles), commentaries, editorials, book reviews, letters to the editor, or where full text was not available. Primary exclusion based on publication focus included those that lacked an exclusive Indigenous focus, and studies with a purely biomedical/disease focus. Other exclusion constituents of publication focus included focusing on old age but not perspectives of ageing well, sole focus on health professional perspectives, and no discussion regarding translation of principles of ageing well into practice.

**Fig. 1:**
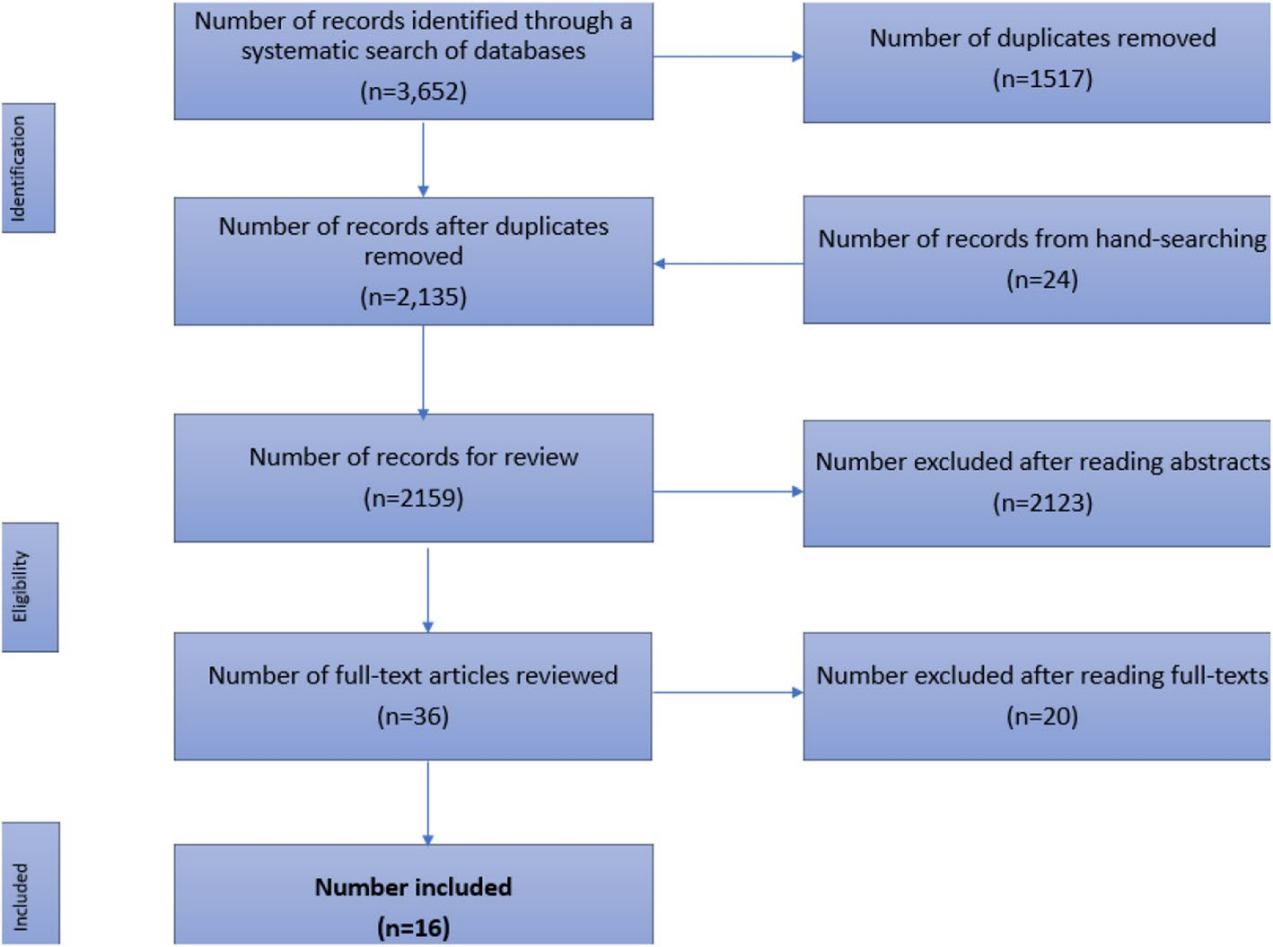
Literature search flow diagram. Excluded as the focus was primarily on developing student capacity rather than the clients [37]. Although an interesting discussion of Elders, it doesn’t address aging well, QoL etc. Wrong outcome [38]. Excluded article type – systematic review [7]. This article was everything relevant to our topic, but didn’t include a specifically Indigenous MoC [27]. An incredibly informative paper with many of the GSGL factors addressed, however didn’t include a specifically Indigenous MoC [39]. Study addresses improving aged care and peoples preferences for care which initiated apparent action. However, didn’t include a MoC [40]. Discussed successful ageing and well-being, however did not mention a MoC [41]. Discussed elder role, and thoroughly addressed well-being. However, a MoC was not included [42]. This paper discussed culturally safe care to Indigenous Elders, however it was Dementia-specific rather than general QoL and well-being [43]. A great reference within this systematic review, but didn’t directly address ageing well even though it does describe preferences [28]. This manuscript had a number of features which met the inclusion criteria and so was referenced in this systematic review, but did not mention a MoC [4]. A terrific paper for gleaning information. Mentioned many specific outcomes. However, the MoC was a work in progress and not established [44]

As with other systematic reviews where there is only a relatively small body of literature investigating the research question, the retrieval of qualitative research is challenging given such factors as limited indexing, non-indicative titles and abstracts [35]. Therefore, conventional systematic review inclusion criteria were broadened to include publications that might otherwise have been omitted (for example reports) [36]. This modification also has the potential to allow for greater inclusion of publications authored by an Indigenous person or including Indigenous people as co-researchers. After the application of these criteria, sixteen publications were identified for inclusion in the review (Fig. 1).

### Synthesis and analysis of included publications through quality assessment, critical appraisal, and GSGL factor alignment

Analysis of the included articles incorporated a decolonizing methodology that privileged the knowledge, cultures, experience and input of older First Nations Australians, their families, carers, communities, and service providers [45]. We undertook three analytic processes: 1. Assessment of quality of articles using the Aboriginal and Torres Strait Islander Quality Assessment Tool (ATSIQAT) (Supplementary Table 4) [46], and the Joanna Briggs Institute (JBI) critical appraisal tool for qualitative research (Supplementary Table 5) [47]. 2. Alignment of reviewed publications with GSGL principles. 3. Thematic analysis of Enablers and Challenges in implementing models of care including the perceptions and experiences of patients, carers, families, and staff/service providers.

### Aboriginal and Torres Strait Islander Quality Assessment Tool (ATSIQAT) analysis

The ATSIQAT tool was developed with the intention of capturing First Nations health narratives according to a First Nations epistemology (Table 1) [46]. Publications that align favorably with the ATSIQAT criteria are thereby seen as offering more validity as findings are interpreted utilizing the context of First Nations values. The ATSIQAT checklist allowed a critical appraisal of the papers that were then graded using a scale of high, medium, or low (Supplementary Table 4). A consensus between authors was established regarding the scaling used to grade alignment of each publication with the ATSIQAT tool. A collective triangulation process between authors DB, EH, and CR quantified the grading process.

**Table 1:** JBI and ATSIQAT assessment criteria

Joanna Briggs Institute items	Aboriginal and Torres Strait Islander Quality Appraisal Tool items
Is there congruity between the stated philosophical perspective and the research methodology?	Did the research respond to a need or priority determined by the community?
Is there congruity between the research methodology and the research question or objectives?	Was community consultation and engagement appropriately inclusive?
Is there congruity between the research methodology and the methods used to collect data?	Did the research have Aboriginal and Torres Strait Islander research leadership?
Is there congruity between the research methodology and the representation and analysis of data?	Did the research have Aboriginal and Torres Strait Islander governance?
Is there congruity between the research methodology and the interpretation of results?	Were local community protocols respected and followed?
Is there a statement locating the researcher culturally or theoretically?	Did the researchers negotiate agreements in regards to rights of access to Aboriginal and Torres Strait Islander peoples’ existing intellectual and cultural property?
Is the influence of the researcher on the research, and vice- versa, addressed?	Did the researchers negotiate agreements to protect Aboriginal and Torres Strait Islander peoples' ownership of intellectual and cultural property created through the research?
Are participants, and their voices, adequately represented?	Did Aboriginal and Torres Strait Islander peoples and communities have control over the collection and management of research materials?
Is the research ethical according to current criteria or, for recent studies, and is there evidence of ethical approval by an appropriate body?	Was the research guided by an Indigenous research paradigm?
Do the conclusions drawn in the research report flow from the analysis, or interpretation, of the data?	Does the research take a strengths-based approach, acknowledging and moving beyond practices that have harmed Aboriginal and Torres Strait peoples in the past?
	Did the researchers plan to and translate the findings into sustainable changes in policy and/or practice?
	Did the research benefit the participants and Aboriginal and Torres Strait Islander communities?
	Did the research demonstrate capacity strengthening for Aboriginal and Torres Strait Islander individuals?
	Did everyone involved in the research have opportunities to learn from each other?

*JBI* Joanna Briggs Institute; Critically assesses the methodological quality of studies to determine the extent to which a study has addressed the possibility of bias in its design, conduct and analysis

*ATSIQAT* Aboriginal and Torres Strait Islander Quality Assessment Tool; Determines the extent to which included articles aligned with First Nations epistemologies

### Joanna Briggs Institute (JBI) Critical Appraisal analysis

The JBI tool for qualitative research critically assesses the methodological quality of studies to determine the extent to which a study has addressed the possibility of bias in its design, conduct and analysis from each tool (Table 1) [47]. These JBI checklists allowed a quality assessment of the papers that were then graded using a scale of high, medium, or low (Supplementary Table 5). A consensus between authors was established regarding the scaling used to grade alignment of each publication with the JBI Critical Appraisal tool. A collective triangulation process between authors DB, EH, and CR quantified the grading process.

### Alignment of reviewed publications with GSGL principles

The 12 strength-based factors of GSGL (Supplementary Table 3) were used to assess the alignment of the reviewed publications by coding the GSGL factors evident in the text. GSGL factors were identified in each publication through a coding process involving authors DB, EH, and CR. A triangulation process between authors DB, EH, and CR ensured consensus regarding the coding process, in addition to quantifying the grading process [48]. Studies were assigned GSGL alignment gradings of high, medium, or low. The twelve domains against which the included studies were assessed were co-designed with Aboriginal Elders and are discussed in detail in Smith et al. [12]. These domains are illustrated in the GSGL framework which can be found in the supplementary material (Supplementary Fig. 4).

### Method for enablers and barrier thematic analysis

The application of a strength-based approach guided the thematic analysis to focus extensively on the enablers, and how they informed a pragmatic approach to delivering appropriate aged-care services to First Nations Elders. Given that in many instances challenges reflect the inverse of an enabler, we only specifically included challenges where they could provide additional insight. An additional analysis of the included articles further demonstrated a First Nations methodology, by focusing on the reported experiences and perceptions of First Nations Elders regarding aged-care service provision.

## Results

The sixteen included publications reported on studies conducted internationally in Australia [29, 36, 49–56], Mexico [57], New Zealand [58–61], and South Africa [62] (Supplementary Table 2). The reviewed studies described either the design, evaluation or implementation of a whole of service model of care design [36, 53, 54, 56–59, 62] or a single service component [29, 50–52, 55, 60, 61]. Twelve of the included articles utilized qualitative methods including interviews and focus groups with three explicitly describing yarning circles – a culturally-appropriate approach facilitating engagement with First Nations people. Two studies that didn’t employ interviews or yarning groups included a peer education intervention and an interpretive research design—were guided using a First Nations methodology in order to accurately capture and represent First Nations’ perspectives [58]. All reviewed studies included qualitative data—including four mixed-methods studies, demonstrating the privileging of participants’ views.

Where information regarding the indigeneity of participants was provided, six publications included both non-First Nations and First Nations participants, with the Non-First Nations participants described as service providers. There were only nine studies which included First Nations participants’ views of services received—an explanation for this relatively low number is the service design focus of the research. All the First Nations participants were Elders with the exception of two studies that included 7 disability clients. Types of care provided were described in context of the setting where provision of care took place – being either home-based, or residential/ respite aged care. Twelve of the publications reported aged-care provision in a home-based setting, suggesting a culturally appropriate approach in the provision of care.

### Models of care

The reviewed publications described specific elements of service delivery (models of care) which may be broadly applied and suitably operationalized to respond to the needs of client/ family in aged care in a culturally appropriate context. As identified in Table 2, publications were categorized as discussing either whole service or individual program/service elements of models of care (see Supplementary Figs. 1–3 for descriptive figures located in the included articles illustrating models of care).

**Table 2:** Overall Summary of Included articles detailing study design and method; information regarding location and context; outline of key findings; and degree of alignment following synthesis and analysis of publications through quality assessment, critical appraisal, and GSGL factor alignment

**Included study**	**Study design and method**	**Sample**	**Context – Urban, Remote or Both**	**Home-based/ Respite/** **Residential aged care**	**Degree of alignment with GSGL**	**Findings related to service delivery, implementing (MOC) principles into practice**	**JBI**	**QAT**
1. Cairns et al. 2022 (QLD) [29]	Single program evaluation; Mixed-methods Action research framework	Unclear	Remote	All	High	Cultural responsiveness is the principle informing service design in remote settings. Co-design enables adaptation to provide meaningful services, improving outcomes. Reciprocal relationships are essential to achieving objectives, and developing strength-based approaches that define good living	H	H
2. Carroll et al. 2010 (WA) [49]	Whole service MoC implementation: Evaluation of a locally designed community service MOC	22 First Nations clients (15 aged care; 7 disability)- aged 15 to 83	Remote	Home-based	High	Recommendations for co-design of integrated aged, disability and mental health service provision, include integrated service coordination facilitated by one culturally safe/community-controlled hub organization working together on common agenda and goals, community-based and culturally secure services, one service access point for Elders, First Nations workforce, and education/ training	H	H
3. Dawson et al. 2021 (SA) [56]	Whole service MoC implementation; case studies Qualitative Interviews	46 members of ACCHO staff & board—20 First Nations Australians, 26 non-First Nations	2 metropolitan Aboriginal Community-controlled Services	All	High	Summarizes principles, enablers, challenges and outcomes of Aboriginal community-controlled aged care service delivery. Details nine implementation actions and service planning activities Sustainability was an issue	H	H
4. Du Toit et al. 2014 (South Africa) [62]	Whole service MoC design; Qualitative Focus groups	15 aged-care workers/ collaborators 8 First Nations, 7 non-First Nations	Urban	Residential	High	Practical suggestions include: meaningful engagement and respect; encompassing autonomy, choice, involvement in co-occupations, and physical accessibility, combined with quality of care	H	M
5. Gidgup et al. 2022 (WA) [55]	Single program evaluation; Qualitative Yarning circles	19 First Nations Elders (program participants) aged over 45	Both – 2 organizations with one from each setting	Unclear	Medium	Learnings related to implementation—describes positive experiences allowing participants to meaningfully connect/ engage in a culturally appropriate program. A sense of belonging was key to attendance and continued participation, with benefits described as holistic in nature, in addition to those derived from the physical activities part of the program	H	H
6. Harding et al. 2022 (NZ) [58]	Whole service MoC design; Interpretive research design guided by Kaupapa Maori theory	17 Health & Social service professionals—12 Maori, 5 non-Maori	Unclear	Unclear	Medium	Key facilitators centered on external support, where participants reported program structure and experiences provide evidence supporting relevance and value of the intervention to their communities. Other key facilitators included process, and adaptability. The process facilitated community engagement during implementation & dissemination- increasing effectiveness & sustainability. Program adaptability enhances cultural/ community fit	H	M
7. Hikaka et al. 2021 a (NZ) [60]	Single program design; Secondary analysis	Data from previous research	Urban	Home-based	Medium	Kaupapa theory underpins five Tiriti O Waitangi principles which structure this pharmacist-facilitated medicines review intervention for community-dwelling Māori Elders, with the objective of achieving equitable changes	M	M
8. Hikaka et al. 2021 b (NZ) [61]	Single program evaluation; Qualitative Interviews	17 First Nations Elders (program participants) aged 58–92	Urban	Home-based	Medium	Recommendations include: medicines knowledge from a trusted professional; increased advocacy; ‘by Māori, for Māori’; increased confidence and control; financial and resource implications	H	H
9. Lavrencic et al. 2021 (NSW) [50]	Single program evaluation; Mixed-methods Yarning circles	7 First Nations Elders (program participants) aged between 62—81	Remote	Home-based	Medium	Co-design process with First Nations communities and stakeholders which privileges First Nations leadership, and is culturally grounded at all stages of design and implementation ensured a high acceptability among participants and facilitators. This enables improved results relating to program objectives	H	M
10. MacKell et al. 2022 (WA, NT, QLD) [51]	Single program design; mixed-methods, online survey, semi-structured interviews & focus groups	99 First Nations and non-First Nations participants—50 artists, 25 art center staff members, 24 aged-care staff	Both	Both	High	Engagement underpinned by connection, Elder role, and culture. MOC is directed by Elders, providing holistic care through meaningful relationships. Reciprocity involves artists working for the center, & community in turn caring for artists	H	M
11. Macniven et al. 2021 (NSW, WA, SA) [63]	Single program evaluation; Qualitative Yarning circles	24 First Nations Elders (program participants)– aged over 45	Both	Both	High	Learnings related to implementation - 6 Key themes around Knowing, Being, & Doing – resulting in a co-design program & resource relevant for Aboriginal people. Knowing involved elders connecting socially to share knowledge – elders value adequate program resourcing, facilities and sustainability. Group dynamics were preferenced, which elders viewed as providing vital social support in order to age well, as well as strengthening culture. Doing involved elders sharing their experiences on operating a culturally-safe, relevant program	M	M
12. Murphy et al. 2010 (WA) [36]	Whole service MoC implementation; Mixed-methods Service data; interviews, journals	22 First Nations clients (15 aged care; 7 disability)) – aged 15 to 83	Remote	Home-based	High	Demonstrated a successful service MOC by incorporating 3 services – disability, older, mental illness—developed in collaboration with community members & key stakeholders addressing unmet needs	M	M
13. Oetzel et al. 2020 (NZ) [59]	Whole service MoC design; Peer education intervention guided by Kaupapa Maori theory	121 Māori Elders	Unclear	Unclear	High	Culturally appropriate peer education intervention can address social connectedness through cultural concepts—tribal identity, *tautoko, whakawhangaungatanga*	H	H
14. Pelcastre—Villafuerte et al. 2017 (Mexico)- [57]	Whole service MoC design; Qualitative Semi-structured interviews & focus group	44 First Nations Elders (aged over 60)– 10 allopathic providers, 10 traditional providers	Remote	Home-based	High	Practical MOC design suggestions made in strategic areas: (1) Sociocultural epidemiology; (2) Local healthcare resources; (3) Community participation; (4) Strategies for communication on health issues; (5) Inter-institutional communication/interaction; (6) Sustainability communication/interaction; and Sustainability	H	M
15. Smith et al. 2010 (NT) [53]	Whole service MoC implementation; Qualitative Participant observation	25–35 First Nations clients aged over 50 mostly frail aged	Remote	All	High	Describes the operating principles ‘cultural comfort’ & ‘community control’ that support Elders to live with family on country. Emphasize community MOC rather than institutional MOC	H	H
16. Wettasinghe et al. 2020 (NSW) [54]	Whole service MoC design; Qualitative Semi-structured interviews & focus group	34 First Nations clients aged 50 years and older	Both	Home-based	High	Practical MOC design suggestions include a culturally-appropriate aged care program which identifies health concerns around themes including physical health, social and emotional well-being, and poor service access. Looks to empower Elders through technology education	M	L

*GSGL* Good Spirit Good Life, *MOC* Models of Care, *JBI* Joanna Briggs Institute, *QAT* Aboriginal and Torres Strait Islander Quality Appraisal Tool, *H* High, *M* Medium, *L* Low, *QLD* Queensland, *WA* Western Australia, *SA* South Australia, *NSW* New South Wales, *NT* Northern Territory, *NZ* New Zealand

### Whole service models of care

Of the nine publications that discussed whole of service models of care, six [29, 36, 49, 53, 54, 57] provided descriptive figures (Supplementary Figs. 1–3). Dawson et al. 2021 proved the most informative of the reviewed publications by providing a ‘process’ for implementing a whole service model of care (Table 3) [56]. The Partnership Service Agreement detailed in Murphy et al. (2010) provides a useful guide to inter-sectorial collaboration [36]. The Cairns et al. (2022) model included the Indigenous Allied Health Australia (IAHA) cultural responsiveness framework principles [29]. Only one publication, (Oetzel et al. 2020) conducted a cost analysis, providing suggestive evidence that their peer education model has the potential to effectively address key health and social outcomes in a cost-effective manner [59].

**Table 3:** Components which contribute to productive management and organization of services as identified by Dawson et al. [56]

**Principles**	**Enablers**
- Respect traditional Aboriginal customs, values & beliefs - Support Aboriginal identity - Connect with elders & community - Culturally safe care - Focus on holistic wellbeing - Respect self-determination - Tailored services - Willingness to go the extra mile - Maintain credibility	- Strong governance - Effective leadership - Organisational culture centred on respect for clients’ Aboriginal identity - Local, caring, qualified & culturally safe aged care workforce - Effective workforce recruitment & training processes - Effective communication - Clear referral pathways - Effective organisational structures & operating systems - Effective financial management systems - Continuous quality improvement processes - Relationships with external organisations
**Challenges**	**Outcomes**
- Funding challenges - Workforce shortages - Change management processes - The need to rapidly develop knowledge of the aged care system - Aged care reforms - Lack of coordination between government departments - Aged care eligibility requirements - Navigating the online aged care portal - Unclear correspondence from social services & government departments	- Promotion of Aboriginal identity & cultural connections - Increased numbers of elders receiving aged care - Reduced pressure & responsibilities on family carers - Improvements in physical health outcomes - Increased access to affordable, culturally safe & quality aged care - Reduced complexities associated with navigating multiple services - Economies of scale - Increased numbers of local, qualified, culturally safe aged care workers

### Single program or service element

The seven publications that described a single program or service element communicated implementation of individual service components in a whole of service delivery model. Examples include, enabling client centered-practice where staff are ‘care partners’ [62]; combining community-based physical activities and yarning sessions [55]; peer education interventions [58, 59]; pharmacist-facilitated medicines reviews [60, 61]; and technology-based health interventions for program delivery [54].

These more targeted research topics provide useful guidance to informing culturally appropriate design and adaption in service delivery. For example, although Hikaka was a ‘medicines review’ intervention, the context in which it was applied required it to be “approached in a holistic manner addressing the domains of wellbeing (physical and mental health, and social connectedness)” [60] (pg. 129). Additionally, resources produced as a part of research projects may provide practical guidance, such as the manual produced by Macniven et al. [63] on conducting yarning circles [52].

### Results of the ATSIQAT analysis and JBI analysis

Using the JBI tool to assess the quality of the qualitative methods, co-authors concluded that there were twelve publications with high alignment. This inferred researchers stated their intentions to conduct research in a manner that faithfully represented First Nations worldviews, while displaying integrity in that their actions replicated the philosophy espoused. Not only were participants voices valued and interpreted appropriately, but they were also published in an ethical manner which demonstrated accountability.

Assessment of the quality of the publications using the ATSIQAT tool concluded there were seven publications with high alignment, eight medium and one low alignment. This suggested that researchers generally displayed accountability towards First Nations communities, as demonstrated by appropriate communication and empowerment involving the projects’ direction and operations. Research was generally assessed as being beneficial towards First Nations communities, with many researchers demonstrating capacity strengthening for First Nations individuals.

No papers were excluded as a result of the quality appraisal analysis. The generally high quality of the reviewed publications adds weight to the strength/validity of findings from the thematic analysis.

### Alignment of reviewed publications with GSGL

Twelve publications measured a high degree of alignment with GSGL principles (Supplementary Table 3), with medium alignments recorded across the remaining four papers. This suggests a strong, general consensus among First Nations Elders and service providers around determining quality of life from a First Nations perspective.

Community, culture, and supports and services represent the most coded GSGL factors following analysis. These factors are explored extensively in the enablers and challenges section of this paper.

### Thematic analysis – enablers and challenges to implementing models of care

Our thematic analysis of the reviewed publications focused on the enablers and challenges to implementing models of care based on GSGL principles. We sought to clearly distinguish these from factors related to the research process. For example, in a fall’s prevention program, decolonizing community-led research had intrinsic benefits that were separate from the program itself [55]. An example of this included creating a culturally safe environment—reassuring and allowing Elders’ to recall and share experiences, for the purpose of establishing reciprocal understanding throughout the research process [55].

In addition, we analyzed the data in the reviewed publications regarding patient/client/Elders experiences of the studied models of care/interventions. These themes relate to enablers including a sense of connection, confidence and being informed and having choices.

### Culture informing First Nations health care—First Nations approach to health

As demonstrated through the GSGL principles, cultural knowledge held by First Nations communities should assist development of and provision of culturally safe and effective care.

First Nations’ views of health are informed by culture and relationships which prioritize social, spiritual, emotional, cultural, and physical well-being [56]. These perspectives are recognized as equally valid to biomedical views, and as epistemologically important within a First Nations context [29]. Therefore, an increasingly holistic approach to health is required, accounting for cultural determinants which support Elder well-being. Underpinning cultural-specific programs with First Nations ways of knowing, being and doing promotes high participation rates, improving quality of life for Elders. Consequently, First Nations ways of working are informed by privileging the voices of Elders [55]. Service provision informed and delivered from within the community allows the cultural construct of the community to inform the framework, including determining relevant health priorities [53].

First Nations ways of knowing, being and doing requires embedding processes which facilitate equal power distribution, highlighting a contrast in First Nations and western paradigms around social dynamics [60]. Two cultural paradigms concurrently informing and operationalizing programs, enhances capacity of First Nations Elders to exercise self-determination—facilitating relevance and feasibility regarding objectives and outcomes [53].

Aged-care service design underpinned by cultural principles, acknowledges and accounts for the roles and lived experiences of Elders. This approach informs meaningful and relevant health priorities [52, 54, 64]. First Nations Elders characterize an ideal Elders’ program as harboring a culturally safe environment, facilitated by proficient staff who support Aboriginal-specific cultural programs involving connection to cultural and community activities [52, 54].

The value that Elders contribute towards their families and communities is recognized by carers as providing an incentive for fulfilling a carers role. “*They are the glue that holds that family together and they are relied upon*” [49] (pg. 19) (Carer 5). “*Even for the little kids they learn a lot off her as well; yeah it’s good for her sometimes. She teaches in languages. Words right and wrong how to respect others, she does a lot of things*.” [49] (pg. 19) (Carer 4). Carers recognized that a lack of culturally appropriate aged care for First Nations Elders is associated with historical trauma relating to institutionalization, a strongly held fear for First Nations Elders. “*She says ‘if I go to them places I’ll die.’ So I think with the family environment, the old girl loved it*” [49] (pg. 21) (Carer 5). This suggests that culturally appropriate aged care involves when possible, allowing families to care for their Elders [49].

However, this dynamic of family involvement in culturally appropriate aged care provision may at times prove detrimental to Elders’ quality of life. While participants reported family members being happy to see their improvements [55], others found that family members can contribute to negative experiences. For example, when family members “*get frustrated with their Elders and sometimes it ends up [with the Elder] being verbally abused, physically abused, or lack of respect for the older person*” [54] (pg. 10). Perhaps reflecting on unsupportive family members, a Māori participant reported that “*being advocated for by someone other than family was the world of difference in my health care*” [65] (pg. 3).

Elder/ community led approaches enhance quality of life by demonstrating specific model of care elements which prioritize healthy relationships. These include distributing burden of care between clients, staff and family members; and promoting intergenerational relationships which allows Elders to engage in mentorship. Country is a term used by First Nations Australians to describe the lands from which their ancestors originated from pre-colonization. Returning there and engaging with communities, presents opportunities to practice and maintain culture by capacitating mentorship. This ultimately addresses social determinants that constitute First Nations perspectives of health [51, 66].

### Connecting with First Nations leadership (Elders) and community to co-design services

The inclusion of First Nations people in robust co-design processes guides development and piloting of programs—informing best practice models of care. Through sharing knowledge and developing mutual understanding, necessary strength-based approaches may be determined [29, 50, 63]. In Gidgup et al. [55] and Hikaka et al. [61] studies the program participants regarded connecting with people and culture as a significant experience bringing positive emotional and physical wellbeing changes. Social connection was seen as more important than other aspects of programs/services—involving strengthening relationships, encouraging involvement, and sharing what they “*had learnt*” in the intervention [52, 59].

Facilitating connection with First Nations leadership (Elders) and communities is achieved through culturally appropriate formal partnerships at each stage of design and implementation, including at the governance and strategic management level. Enablers to engagement and good communication include: privileging First Nations voices and acknowledging leadership [55]; non-First Nations service providers employing an empathic approach to build respect and maintain credibility with clients, families and community [56]; providing cultural safety training for all staff [36, 52, 56]; using culturally appropriate communication such as yarning (circles), employing a compatible approach to address concerns of staff and clients [36, 50, 54, 55]; creating a comfortable space [55]; engaging independent facilitators [36] and mentors including past program participants to inform current participants and staff on the most effective methods of operationalization [58].

The ability to form connections with First Nations Elders/people involves identifying and appropriately navigating the barriers which negate effective facilitation of connection. Recognized barriers include feelings of shame when requiring help and experiencing a sense of hopelessness or disempowerment regarding ageing well [54]. With regards to service providers, barriers identified included frustration when attempting to facilitate connection or build rapport—significantly associated with remote settings [36].

An approach to alleviating the recognized shame and hopelessness barriers involves implementing group dynamics. A diverse constituent of First Nations community members comprising board membership, provides integral connection with communities to inform program development, particularly where there are strong relationships between board, management and staff [29, 56]. Similarly, the reviewed literature suggests stakeholder groups which demonstrate a permanent presence within their communities facilitate greater connection [29]], sustaining success in cross-cultural settings through initiation and accommodation of change over time [53]. Engagement in service co-design processes is beneficial to Elders as it promotes and strengthens self-determination, allowing Elders to learn and expand their knowledge about the requirements of delivering aged-care services. Furthermore, engagement promotes cultural identity and well-being, by acknowledging and implementing meaningful and relevant experiences and understanding [55, 59, 62].

Aboriginal Community Controlled Organizations (ACCOs) provide an organizational culture that promotes respect for clients’ cultural identity. As such, they are often best placed to accomplish the aforementioned objectives. Their connection to Elders and communities ideally positions them to tailor services to needs and address social issues in order to increase self-determination [56].

### Supports and services must be based in co-design principles and planned for

Following engagement with consumers and stakeholders, systemic planning of health services is necessary in order to operationalize co-design principles [60]. At a service delivery level, reviewed publications described specific elements of service delivery (models of care) which may be broadly applied and suitably operationalized to respond to client/ family needs in a culturally appropriate context. Service delivery elements were analyzed as enablers related to two subthemes—the internal service operation enablers and those related to the provision of services to Elders.

### Organizational level enablers

Dawson et al. [56] provides a comprehensive list of components that contribute to productive management and operationalization of services (Table 3).

Dawson et al. [56] also focused on the importance of prior planning, including consultation with Elders, mapping existing aged care services and workforce, visiting an ACCHO already providing aged-care services, desktop auditing, and a scoping review. Another example from the literature recommends that these organizational level enablers converge to create a single point of access for service enquiries, increasing efficiency of navigation and access to multiple services for clients [36]. Carroll et al. [49] co-designed an integrated model of community care following community development principles with remote community members, caregivers and services across a remote region of Western Australia. This model was then funded by the integrated services including aged care, mental health and disability organizations for a 12-month trial with one remote First Nations community. Key components included the development of a community led common agenda with measurable goals, a culturally safe organization (e.g., community controlled) facilitating a community led service partnership, and First Nations community council representatives at the governance level. The role of external government services changed from direct service delivery to training and capacity building of a community-based Aboriginal workforce for in-home care. Finally, one of the reviewed publications also provided examples of communication channels between intercultural promoters and national health systems, facilitating the creation of increasingly elaborate and collaborative networks among health system [57]. This also extends to respective service providers—negating frustrations around lack of clear referral pathways, while promoting general consensus regarding best care practices across departments [49].

### Enablers related to responding to client/family needs

Ensuring a favorable balance between cultural considerations and service operations involves developing and delivering on common community and client directed agendas and objectives [59]. This was a key outcome of Lungurra Ngoora model [49].

Effective service delivery entails supporting caregivers, including liaising to arrange the appropriate supports. “*Caring can place huge pressure on carers, I’ve got four kids of my own plus um one of my sister’s child I’m looking after as well. It’s a big job, it is yeah trying to work and juggle family at the same time…(and) she’s like a big kid herself, it’s very hard*”. [49] (pg. 20) (Carer 4). Equally, service providers also alleviate any frustrations they may have experienced in their role when engaging with carers, as clear and effective communication ensures they become aware of and are able to deliver on carers’ needs [49].

An approach which strengthens connection to family and community in aged care, should additionally focus on clients’ cultural values, strengths [62]. This enhances cultural values such as respect for Elders, ensuring caregiving retains human dignity despite physical or cognitive deterioration [62]. A feature of programs operating in this space is that they must be flexible in applying a cultural model of interest as determined by the community [54, 58, 60, 61]. This enhances culturally responsive care [29].

While an Indigenous workforce is preferred, the first change to a model of care may involve community service delivery that accommodates care providers working alongside First Nations aged and disability support workers during home visits [29]. Advocacy embodies the essence of culturally-principled aged-care provision to First Nations Elders, empowering communication with other health professionals, while facilitating access and service navigation [61]. Advocacy also promotes communication between family carers and service providers; and assists staff members delivering education to the community [36].

Empowerment of First Nations Elders to further communicate their health perspectives and priorities can be facilitated by holding educational forums or expos that enable those accessing services to share experiences with peers and service providers. Doing so creates a reciprocal dialogue around topics including successful ageing [59]. Information collected from these forums can be translated into relevant and meaningful information deemed culturally and linguistically appropriate as it responds to communities’ needs, with dissemination of the material through community sources [57].

Strong communication with health care providers [61], while being informed and provided with choices, is important for Elders to feel confident and in control of their own lives [62]. Supporting Elders to take self-determined action enhances their cultural identity and wellbeing, and realize their full potential “*to think differently, with new hope, attitude and understanding*” [59] (pg. 8). In this way it was acknowledged “*that you really have to take time for yourself”* [52] (p.4) and view participation in programs as “*self-giving*” [59].

Improved access to information was strongly associated with improved wellbeing. Wettasinghe et al. [54] reported that participants expressed a desire for health education, arguing that this supported participants to make informed health and lifestyle decisions. This mitigated feelings of hopelessness and resignation toward health issues associated with ageing.

Social groups enable knowledge sharing between Elders, providing a platform for Elders to express their desires and concerns, while being informed on how to effectively address their health and wellbeing priorities. A healthy lifestyle group participant from Macniven et al. reported that:

‘*It’d be you’d have to have your group saying what they want to know about. Could be health could be all of that, but led from the group … we’ve all got different skills. So it’s nice to be able to have somewhere where we can pass those skills on’* [52] (p. 3).

Particularly highly valued was strengthened access to information about “resources” and “services”, as well as “people” who could help [59].

The way information is conveyed is particularly important. For example, *“[The pharmacist is] Māori; she can communicate in a way that enhances my* mana *(prestige, standing, authority*)” [60] (pg. 4). It is important to be able to talk to someone knowledgeable and to feel able “*to ask the things I wanted”* [60] (pg. 3).

As previously mentioned, culturally appropriate means of communication and relations represent the most effective means by which to operationalize program service delivery components, both internally within the organizational structure, and externally from service providers to patients. This involves utilizing a clinical yarning approach when communicating in a clinical setting [29].

Historically, First Nations perspectives on institutions such as residential aged care have been negative as they are regarded as places people go to die [56]. Employment of First Nations staff and clear and appropriate communication may assist in reducing fears about residential care, particularly for Elders with trauma from being forcibly removed from family and into institutional care [56]. “*It’s about them probably knowing this is an Aboriginal organisation and Aboriginal people work here”* [56] (pg. 3).

### Development of workforce

Following on from systemic planning of health services, an appropriately trained workforce is required to operationalize service delivery. The reviewed literature specified the requirements relating to hiring and training of staff delivering services to First Nations Elders. Employment of staff analyzed in accordance with enablers, related to two subthemes—staff from community or First Nations staff, and supporting staff external to community.

### Staff from community or First Nations staff

The reviewed literature frequently mentioned that best practice involved hiring of local community members, or First Nations staff to provide care to Elders. These are recognized as key resources who potentially represent great advocates for clients, further reinforcing engagement by clients and families [36, 56]. Community development practices of support and upskilling of local populations enables continued refinement of service efficacy [53]. The need for an Aboriginal workforce was emphasized, both to better understand patient needs, and to prevent Elders from feeling shame at being cared for by a stranger [49].

A key objective highlighted in the literature is to increase numbers of community-based Aboriginal staff in remote communities who are paid a salary, rather than relying on top up funding with government support payments [36]. Successful employment of First Nations staff includes employing and supporting senior First Nations representatives from the community to fulfil strategic management and board roles of the service [36, 60, 61]. Additionally, pools of casual workers allow continuation of day to day service provision with minimal disruption, covering unforeseen circumstances such as cultural business affecting staff attendance [53]. Applying the above incentives enables programs to continue functioning despite staff turnover, with senior community based management and board members identifying community members who could fill the roles [36]. Ultimately, building self-determination amongst local staff members is a cultural principle upon which services are founded—reinforcing local ownership, independence and providing culturally appropriate space to facilitate and support local workers [53].

Supporting First Nations staff involves providing assistance with regards to workload and delivering on culturally based principles as staff members. These areas are described in the literature as contributing to frustration, and resignation when they are not recognized and handled diligently [29].

Supporting and developing the capabilities of community staff members, is essential to optimizing quality of life in First Nations Elders. An external reviewer noted that “*feedback from participants is very positive and suggests that their quality of life, participation and contribution in their local community has been enhanced… providing a greater quality of life to clients and the … community as a whole*” [36] (pg 20–22). Additionally, First Nations staff reflected improvements in their own wellbeing, due to increases in local training and employment opportunities [56]. Another important factor is the recognition of the “*relationship between the management … [Aboriginal staff members] and the Board is probably one of the strongest parts of the organisation’s operations*” [56] (pg. 5).

### Supporting staff external to community

Training and development are required in order to build the capacity of external staff providers to deliver culturally appropriate care to First Nations Elders. Providing adequate clinical and cultural supervision, as well as adapting clinical processes to support culturally responsive care ensures this [29]. As discussed earlier, it is imperative to offer training specifically in First Nations aged care, when an organizations underlying principles and culture is based on respect for clients’ cultural identity [56].

An ideal culturally safe and effective aged care workforce consists of First Nations staff members who are appropriately trained, understand the aged care and health system, bilingual in regions where necessary, and possess a cultural worldview to liaise effectively between traditional and biomedical health domains [67]. This starts with best practice aged care education and training for First Nations health and aged care students. Cairns et al. discussed how an inter-professional aged care service delivery model in a remote community allowed for discipline-specific training and supervision to students during community placement [29].

As previously stated, operationalizing service delivery requires flexibility in time management, and matching both First Nations and non-First Nations workers needs with those of the service. This involves finding culturally sustainable ways of meeting client and workers’ needs [53].

Worker’s satisfaction is correlated with best practice aged-care to First Nations Elders that promotes control and empowerment. Non-Indigenous service providers working within a community controlled organization reported satisfaction from building empowering relationships with Elders [56, 62].

“*Don’t make decisions for them, ask them. And I know that it’s – it’s talked about a lot, and it doesn’t happen often enough. I think that’s something people could learn from us*” [56] (non-Indigenous ACCO staff member Dawson et al. 2021) (pg. 4).

### Funding and resourcing

Successful funding applications, including recurrent funding which support infrastructure of new positions, are crucial to increasing program effectiveness [29]. Identifying appropriate funding bodies and accountable allocation of funds ensures operations and management of services can be supported and sustained in providing appropriate quality of care to First Nations Elders. Accessing multiple revenue streams is often necessary to coordinate and deliver programs, requiring reporting to government bodies, land councils, in response to receiving donations, and self-funding [52].

Sustained funding is important in mitigating negative experiences reported by Elders – such as the disappointment felt when funding finishes and the service ends, or feeling enticed to join a program that fails to deliver on its objectives [52]. “…*they say come and join this program, we’re going to help you with this, this and this [*then*] Sorry we can only give you this*” [52] (pg. 4).

Further to this, the capacity of the workforce to operate is contingent upon provision of quality resourcing which includes promoting environments conducive to both staff and clients, encompassing provision and transaction of supplies and services [54]. This premise was exemplified in the literature by detailing how limitations impacting resources and funding compounds the physical limitations affecting Elders by impacting on visits to country [51], or lack of counselling for grief and loss, especially after putting a partner into care [52].

Issues around funding were identified as a consistent barrier. Models of care that addressed issues at the service level were often reported as being associated with new problems for services in relation to resourcing. The clearest example of this was reported in Dawson et al. [56] where one of the study sites went into financial deficit as a result of the process of integrating aged care services. Dawson et al. (2021) endeavored to provide services at no out of pocket expense to clients [56]. Faced with the same predicament, Cairns et al. [29] proceeded to employ existing resources to fund programs once revenue allocation had been exhausted – requiring the Chief Executive Officer (CEO) to function in a voluntary capacity.

Efficient management of funding requires a balanced approach which accounts for objectives determined by funding bodies, and the priorities of communities/ programs—ensuring equal distribution of power which delivers timely solutions [58]. Oetzel et al. [59] was the only publication to conduct a cost analysis, finding that their peer education model for aged care had the potential economically to effectively address key outcomes.

## Discussion

The importance of First Nations Elders to the health and wellbeing of communities provides an important driver for developing culturally appropriate in home and residential aged-care models. The sixteen reviewed studies provided valuable guidance to inform best practice models of aged care for First Nations people – informing the design, implementation and evaluation of whole of service models of care or a single program or service element. Analysis of the quality of publications using the ATSIQAT and JBI critical appraisal tools demonstrated general alignment with the strengths and values of First Nations communities and good qualitative research practice. This suggested that findings are generalizable and applicable. Additionally, the significant overlaps between identified enablers and GSGL factors is validating and reflects a broad consensus among First Nations elders and service providers regarding healthy ageing factors from a First Nations perspective. All studies incorporated some of the twelve GSGL quality of life factors important to First Nations Elders (range of 5–12 factors) (Supplementary Table 3). This suggests the importance of these components to First Nations Elders care. The three factors that were most frequently incorporated into studies were community, culture, and services and supports. Only six of the studies discussed spirituality in their research. This factor is of particular important to First Nations Elders as they age [11, 12]. More research is required on how to suitably operationalize and embed spirituality and the other eight GSGL factors into aged care. Through our review process, we identified a number of systematic reviews related to best practice care in other health domains [20, 21, 68]. These publications further support our review findings and the GSGL research, whilst adding weight to the need for system change. Operationalizing Elder co-designed aged care principles (such as the GSGL factors) to create changes within health and aged care organizations, has been mandated by high level policy and government change through the Royal Commission into Aged Care Quality and Safety [5]. Our discussion focuses on the detail of the system changes/service delivery/ change management required within a broader decolonizing approach—including workforce, funding, and partnering outside of the health sector.

### Colonisation

First Nations people have historically been disenfranchised through colonization, involving implementation of institutional policies and practices intended to extinguish self-determination. A paternalistic endeavor, this is generally considered to have detrimentally impacted First Nations’ health due to invalidation of Indigenous knowledge systems [56, 69]. Residual effects of Colonization manifest in contemporary times through deficits in socio-cultural indicators [70]. These inequalities in social and health indicators for First Nations Australians have ultimately equated to lower life expectancy and increased burden of disease [70].

Western perspectives characterizing health, well-being and quality of life have traditionally emphasized the individual, whereas First Nations conceptualization accounts for the social, emotional and cultural well-being of, and connection to the community [70]. These factors are not always considered in health and aged care policy and program development [7]. The reviewed studies were conducted in four countries that accommodate considerable First Nations populations and are historically associated with colonialism.

A notable omission in the reviewed publications is North American countries—in particular Canada, which not only has a close affiliation with Australia as a Commonwealth nation [71], but also has a history of colonization whereby First Nations populations have been assimilated under Eurocentric approaches to policy making [15]. It has been suggested that the paucity of publications that prioritize implementation of Canadian First Nations Elders’ perspectives into service delivery [72], is a reflection of the political landscape in Canada. Canada has recently been dominated by the Truth and Reconciliation Commission of Canada 2015, which prioritized the need for redress concerning the residential schools [73]. While this affects the quality of life and well-being of Canadian First Nations Elders, it doesn’t directly address service delivery related to ageing well from a First Nations perspective, thus aged care remains largely settler-centric [73]. Contrastingly, the Royal Commission into Aged Care Quality and Safety in Australia has provided an important policy driver concerning aged-care service structure and delivery to First Nations Elders. Reform would be accomplished through translation of First Nations knowledge, into practical solutions to increase quality of life for First Nations Elders [74].

### System change/Change management

A decolonizing approach to First Nations health requires systematic changes at every level [75]. Knowledge translation will require service structures and systems to be organized in a different way, including service delivery, as exemplified by the work of Wright et al. [21, 76, 77].

Privileging the voices of First Nations elders facilitates translation of principles (such as those presented in this review) into ways of working [17]. This results in implementation of models of care that include a more comprehensive understanding of health, rather than an exclusively clinical approach [75]. These enablers inform a change management process which is required in order to achieve a top-down systematic or “decolonizing” change within First Nations aged care service.

Change management processes must allow for a systematic change to services, and influence every aspect of service design, structure, and delivery in addition to funding allocation and workforce. It is not sufficient to enact change management processes at a single level of service delivery. A top-down, systematic approach to change management ensures congruency begins with principles, guiding every aspect of the service from co-design to service delivery.

Change management processes require much greater time spent with clients and their families than typically afforded to allied health professionals working in remote communities, and much longer than most project or research funding allows. Short-term funding initiatives do not usually support the time required for this work. For example, Dawson et al. discussed the challenge of change management processes in relation to integrating aged care within the Aboriginal Community Controlled Health Organization’s (ACCHO) primary health care service delivery model (e.g. communicating with staff, establishing referral pathways) [56].

**Fig. 2:**
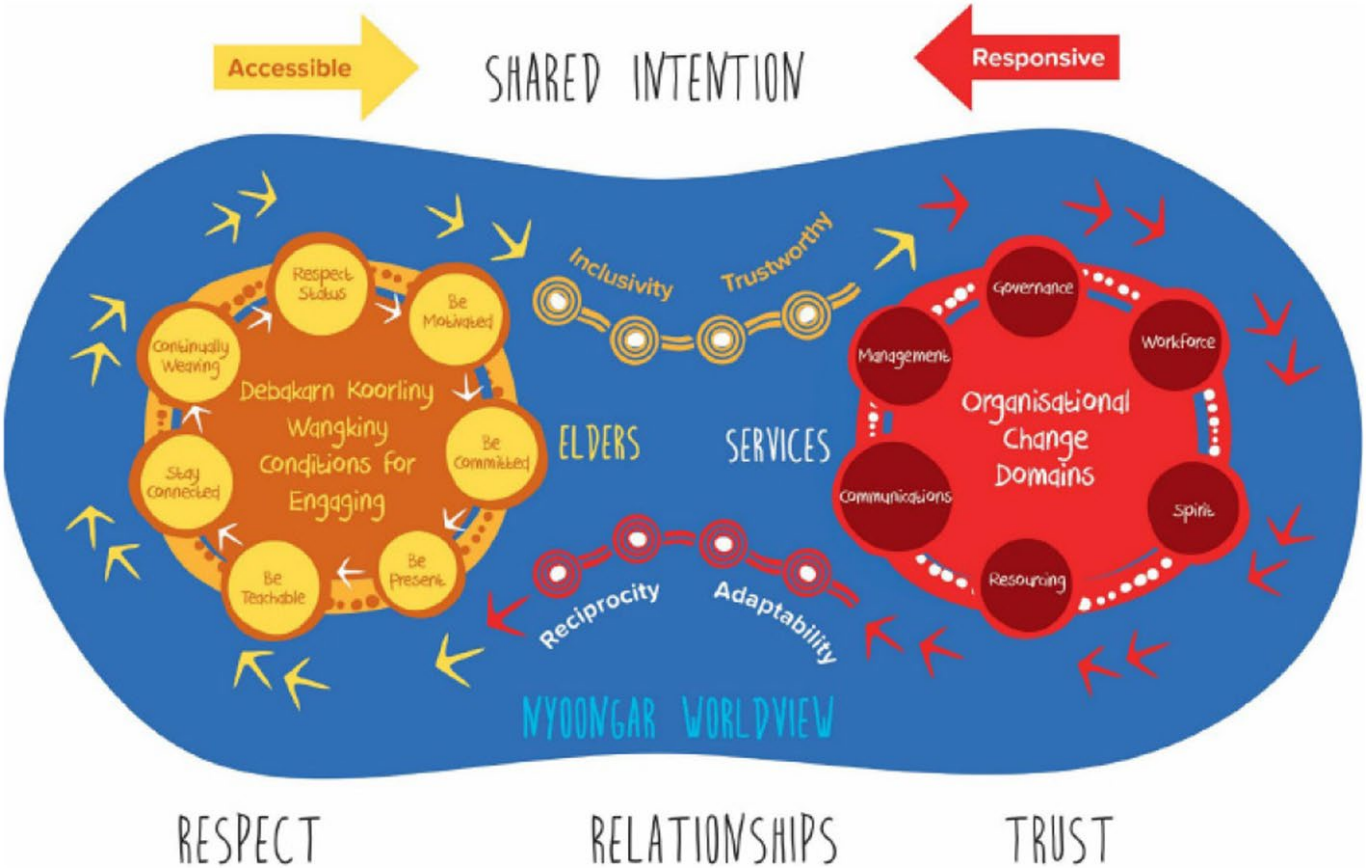
The *Minditj Kaart Moorditj Kaart* Framework for Systems Change detailed in Wright et al. [76]

With a focus on improving mental health and drug and alcohol outcomes for Aboriginal people, the work of Wright et al. [21, 77], is outside the review inclusion criteria. However, they provide a strong example of knowledge translation actions for systems change based on Aboriginal priorities. The *Minditj Kaart Moorditj Kaart* Framework for Systems Change [76] (see Fig. 2) can be applied to implement a model of care based in principles such as GSGL principles. The implementation framework was co-designed by Aboriginal Elders and includes the principles of *debakarn koorliny wangkiny* (steady walking and talking) for engagement, the four principles of relationship-building, and the Burdiya to Burdiya first principle of working together.

### Workforce and staff training

As part of the systematic reform of aged care services, establishment of an appropriately trained aged care workforce is required in order to operationalize service delivery. This requires staff to be educated and informed around working with First Nations communities [37]. For example, cultural safety training informs workers, but guides organizations in understanding cultural principles (including a holistic health definition and its determinants) and operationalizing these models of service delivery.

Highly valued throughout the literature was hiring of local community, or First Nations staff to provide the services to elders. Community-based staff, including local First Nations community members are recognized as key resources who potentially represent great advocates for clients, further reinforcing engagement by clients and families [36, 56]. However, organizations with limited First Nations workforce need to be aware of not placing additional burdens on First Nations staff by asking them to provide cultural knowledge, but rather look to other sources such as a board comprised of community representation. A significant reciprocal benefit is that providing better care can increase workforce satisfaction and improve staff retention.

### Looking beyond health services

Many of the reviewed publications provided strong support for collaborations/partnerships with other service providers to support changing models of service delivery within health services. This aligns with Royal Commission into Aged Care Quality and Safety recommendations system reform that prioritizes integrated models of care. Similarly, calls by Indigenous scholars for system reform advocates for a fundamental shift from biomedical and siloed models of care, to integrated models that centralize cultural expression and safety, holistic definitions of health, prioritize connection to Country, intergenerational connection and the cultural determinants of health.

Mackell et al. [51] demonstrates that art centers have potential to play a significant role in supporting the holistic health and wellbeing of older people, who are often the cultural pillars of the art center in remote First Nations communities. These findings are supported by Wettasinghe et al. [54] who note that participation in arts programs can improve physical and mental health and well-being, foster greater social cohesion, and support cultural identity. Arts centers are not bound by siloed funding, and so are positioned to provide a wide range of holistic care, assistance with navigating health and aged care systems, as well as collaborating with aged care and health providers [51].

Mackell et al. [51] suggests that the benefits of integrating shared models of care between aged care and art centers include: sharing scarce infrastructure; reducing stigma associated with accessing aged care services; and promoting belonging and integration for all generations. Arts centers represent organizations that are closely aligned with First Nations principles, providing culturally safe environments which promote wellbeing as they address cultural determinants of health. Mackell et al. [51] emphasized the role of arts center staff who reported that they typically have better relationships with Elderly artists than staff in the health centers and can get more done for them as a result. Particularly their knowledge of other service providers enabled them in helping people to communicate with or access health services. Arts center staff were able to gain unique insights into the health, wellbeing and social situation of their older artists through regular contact with artists and the ability to notice changes physically and cognitively.

Arts centers represent the type of organization that would benefit health services to integrate with, as they are more fluid in their service delivery and funding capabilities. Further exploration about how art centers could be recognized, resourced, and implemented within Australian aged care, health and disability policy frameworks is warranted.

Another important example of looking beyond health services to provide aged care (Supplementary Fig. 1), Smith et al. [53], clearly articulates a model based in community strengths.

*“In this way the local organization and local workers are enabled to work with families and to care for old people in a flexible and culturally appropriate way. It also means assisting families to maintain traditional ways of caring for older family members while providing new support services to complement and sustain traditional patterns of care*” [53] (pg. 12).

Working with community should include capacity building in research and data to enable participatory roles in defining their health priorities through sociocultural epidemiology. Similarly, capacity for community involvement in action planning also involves health surveillance, administration of resources, and their own provision of services [57]. Such collaborations/partnerships need to be based in strong Inter-institutional interaction, collaborative networks among the health system, and local and civic service organizations [57]. For a detailed example see the Partnership Service Agreement provided by Murphy [36] as a useful guide to inter-sectorial collaboration.

Our findings are broadly consistent/in agreement with a recent international systematic review [7]. They explored strategies by which health and aged care services can support the well-being of older First Nations people. Findings were grouped into 3 key themes: maintaining Indigenous identities; promoting independence; and delivering culturally appropriate care. These can be realized by maintaining important relationships with family and communities, and providing opportunities that enable older First Nations people to connect with and support one another, while also maintaining a connection to land and country and supporting traditional ways of living where possible. Elders should be encouraged to remain physically active and to continue to contribute to their communities, such as through acting as custodians for traditional knowledge and passing this on to younger generations. Importantly, health and age care services must be culturally safe places, which First Nations Elders should have a role in the design and management of [78].

### Funding

Despite evidence verifying the benefits of instituting new approaches to First Nations aged care in service design and implementation, securing funding presents a critical area of focus relating to the long-term success of service delivery [56]. Reasons for the inability to sustain financial support may be varied and complex, such as securing long-term funding where programs are trailed in various locations [79].

In addition to this, an inconsistent political environment where governments may change numerous times within a ten-year funding agreement, presents complicated conditions to navigate [79]. Additionally, as programs and services must be administered in accordance with objectives stipulated by funding bodies it may be necessary to negotiate new agreements between services and funding bodies in terms of reporting and measures.

### Strengths and limitations

We attempted to reduce the risk of bias in our review process by ensuring that each study included in the review was assessed by all members of the research team, who had diverse personal and research backgrounds.

This systematic review was informed by the GSGL framework, which is co-designed by First Nations Elders and privileges their worldview, exemplifying a decolonized approach to First Nations Elders QoL. This satisfies a major policy imperative in the form of The Royal Commission into Aged Care Quality and Safety, which identified an urgent need to plan for culturally safe care of older First Nations people in Australia.

This systematic review included only English language articles. This was an unavoidable limitation given the languages spoken by the research team. However, this might mean that potentially important information was not reviewed. For example, information about First Nations groups in Canada (such as Métis) may be reported in the French language. Similarly, this review only considered original research, and as such, some grey literature that was not picked up by our search strategy, but was reviewed in other reviews, may not have been included.

## Conclusions

First nations Elders are an important source of cultural strength and knowledge for their communities. Colonization has adversely impacted the health of First Nations populations around the world, and this continues to be perpetuated by systematic means including lack of appropriate service provision [3]. The Royal Commission into Aged Care Quality and Safety highlighted an urgent need to plan for culturally safe care to meet the needs of the projected number of older First Nations people in Australia [5, 6]. Many are likely to be part of the historically marginalized and potentially institutionally traumatized Stolen Generation members (who will meet the age criteria for aged care services by 2023) [5, 6, 10].

Our review sought to provide support for services intending to implement decolonizing approaches such as those described by GSGL, to aged care by identifying enablers to providing best practice models of care for First Nations Elders. While there are significant examples of co-design processes which have developed principles to guide culturally safe and appropriate delivery of services [21] enacting these principles needs to include a systems level approach which guides the way services and interventions are delivered on an individual level. That is, as well as tailoring supports and services to meet the needs of the community, organizational priorities must include developing an effective workforce and identifying appropriate partnerships outside the health domain.

This decolonizing approach privileges the voice of First Nations communities, facilitating translation of principles into ways of working, promoting holistic quality of life and inner spirit for First Nations people [80].

### Supplementary Information

**Supplementary material 1.**

## Data Availability

All data generated or analyzed during this study are included in this published article and its supplementary information file. Data and associated materials detailed in this systematic review are not publicly available.

## Abbreviations

GSGL: Good Spirit Good Life
ATSIQAT: Aboriginal and Torres Strait Islander Quality Assessment Tool
JBI: Joanna Briggs Institute
MOC: Model of Care
IAHA: Indigenous Allied Health Australia
ACCO: Aboriginal Community Controlled Organization
ACCHO: Aboriginal Community Controlled Health Organization
CEO: Chief Executive Officer
NZ: New Zealand
QLD: Queensland
NSW: New South Wales
WA: Western Australia
SA: South Australia
NT: Northern Territory
