# Supplementary material for: Best practice models of aged-care implemented for First Nations people: a systematic review aligned with the Good Spirit Good Life quality of life principles
Source: BMC Geriatr. 2024 Feb 29;24:210. doi: 10.1186/s12877-024-04781-0 (PMC10905862; doi:10.1186/s12877-024-04781-0)
Supplement: Supplementary file 1 — Supplementary material 1. [file 12877_2024_4781_MOESM1_ESM.docx]

### **Supplementary Table 1. Search String**


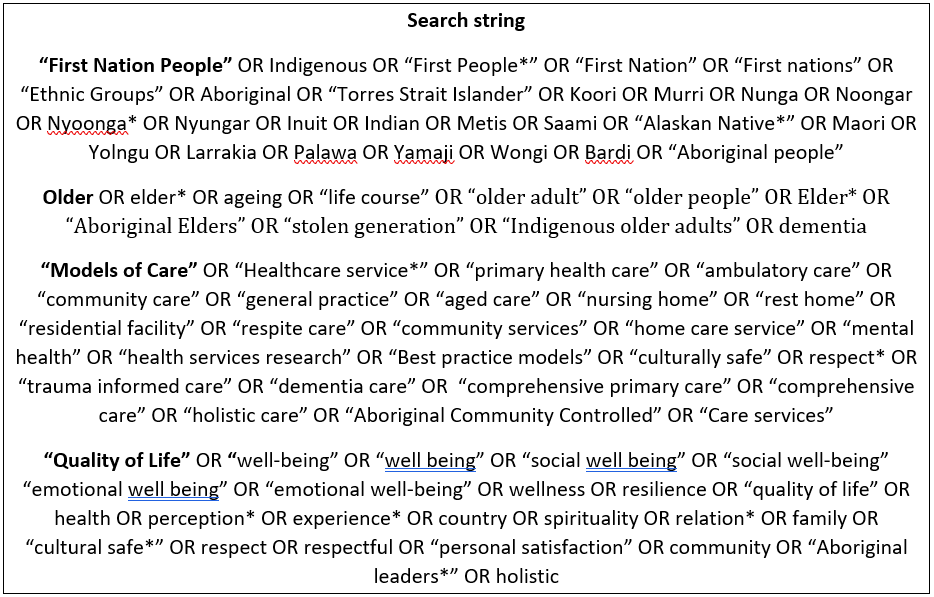


### **Supplementary Table 2. Inclusion/Exclusion Criteria**

| Criterion | Inclusion | Exclusion |
| --- | --- | --- |
| Publication focus | Perceptions of, attitudes to, concepts of, cultural aspects of, definitions of, aging well and associated terms.  Discussion of domains of aging well.  Implementation of principles of ageing well into practice.  Care provision in either residential or institutional care.  Evidence related to implementation of culturally appropriate care based in community-identified quality of life factors, can be whole service delivery or single program only  *Concerned with strategies to improve quality of care based on patient, family, and carer satisfaction.* | Perspectives of aging well (and associated concepts) or measures of aging well of First Nations peoples that were incorporated into wider cultural groups.  Focus on specific diseases of aging. Focus on cellular or biological aging. Focus on older age but not perspectives of aging well (or associated terms).  Focused solely on the  perspectives of health professionals.  No discussion of implementation of principles of ageing well into practice. |
| Population | Older First nations peoples worldwide and their families and carers |  |
| Language | Published in English |  |
|  | Original research including qualitative, quantitative and mixed methods*.  Grey literature, government, peak bodies or organizational reports, website information. Full text available. | Literature reviews (relevant articles from these included), commentaries, editorials, book reviews, letters to the editor, or where the full text was not available. |

* Given the focus on attitudes, beliefs, expectations, understandings, perceptions and experiences, this systematic review focused on qualitative studies, including qualitative findings within mixed method, case study and case series studies

### **Supplementary Table 3. Alignment of Reviewed Articles with GSGL Factors**

|  | **1** | **2** | **3** | **4** | **5** | **6** | **7** | **8** | **9** | **10** | **11** | **12** | **Total** | **Alingment** |
| --- | --- | --- | --- | --- | --- | --- | --- | --- | --- | --- | --- | --- | --- | --- |
| **1. Cairns et al. 2022 (QLD)** | 1 | 0 | 9 | 10 | 2 | 2 | 0 | 11 | 2 | 0 | 3 | 1 | 9 | H |
| **2. Carol et al. 2012 (WA)** | 7 | 3 | 10 | 3 | 3 | 0 | 1 | 17 | 0 | 1 | 0 | 5 | 9 | H |
| **3. Dawson et al. 2021 (SA)** | 1 | 1 | 4 | 4 | 1 | 2 | 1 | 12 | 0 | 0 | 2 | 0 | 9 | H |
| **4. Du Toit et al. 2014 (South Africa)** | 1 | 0 | 4 | 5 | 1 | 2 | 1 | 3 | 1 | 0 | 0 | 3 | 9 | H |
| **5. Gidgup et al. 2022 (WA)** | 2 | 0 | 2 | 3 | 4 | 3 | 0 | 1 | 3 | 0 | 0 | 0 | 7 | M |
| **6. Harding et al. 2022 (NZ)** | 1 | 0 | 4 | 4 | 0 | 1 | 0 | 0 | 0 | 0 | 1 | 1 | 6 | M |
| **7. Hikaka et al. 2021 a (NZ)** | 1 | 0 | 0 | 8 | 3 | 3 | 0 | 3 | 2 | 0 | 6 | 1 | 8 | M |
| **8. Hikaka et al. 2021 b (NZ)** |  | 0 | 0 | 2 | 1 | 0 | 1 | 4 | 0 | 0 | 3 | 0 | 5 | M |
| **9. Lavrencic et al. 2021 (NSW)** | 2 | 1 | 1 | 6 | 4 | 0 | 4 | 7 | 3 | 1 | 3 | 3 | 11 | H |
| **10. Mackell et al. 2022 (WA, NT, QLD)** | 2 | 1 | 1 | 6 | 4 | 0 | 4 | 7 | 3 | 1 | 3 | 3 | 11 | H |
| **11. Macnivnen et al. 2021 (NSW, WA, SA)** | 2 | 2 | 8 | 7 | 4 | 2 | 2 | 5 | 2 | 0 | 0 | 0 | 9 | H |
| **12. Murphy et al. 2012 (WA)** | 5 | 2 | 5 | 7 | 3 | 1 | 0 | 15 | 2 | 0 | 1 | 4 | 10 | H |
| **13. Oetzel et al. 2020 (NZ)** | 2 | 2 | 6 | 11 | 7 | 1 | 1 | 7 | 0 | 1 | 1 | 3 | 11 | H |
| **14. Pelcastre et al. 2016 (Mexico)** | 1 | 3 | 3 | 4 | 7 | 1 | 1 | 2 | 0 | 2 | 1 | 1 | 11 | H |
| **15. Smith, Grundy & Nelson 2010 (NT)** | 9 | 5 | 20 | 21 | 3 | 0 | 1 | 15 | 5 | 0 | 1 | 5 | 10 | H |
| **16. Wettasinghe et al. 2020 (NSW)** | 6 | 0 | 13 | 15 | 13 | 4 | 5 | 3 | 8 | 0 | 3 | 0 | 9 | H |
| **Totals** | 44 | 21 | 90 | 123 | 65 | 23 | 24 | 111 | 31 | 6 | 33 | 30 | 152 |  |

GSGL Factors – 1. Family and friends, 2. Country, 3. Community, 4. Culture, 5. Health, 6. Respect, 7. Elder role, 8. Supports and services, 9. Safety and security, 10. Spirituality, 11. Future Planning, 12. Basic needs

The top row of Supplementary Table 3 displays a chromatic scaling numbering 1 to 12 in bold numbering, representing the GSGL Factors as detailed in the note section under the table. The far-left column displays a vertical sequence of names identifying the included studies. The numbers in each cell quantify the instances identified for each GSGL factor within each publication. The bottom row of Supplementary Table 3 displays the overall number of instances each GSGL factor is identified throughout all included publications. The far-right column represents the degree of alignment with the GSGL factors that each publication displayed. Studies were assigned GSGL alignment gradings of high, medium, or low, quantified as High = 9-12, Medium = 5-8, Low = 1-4.

**Supplementary Table 4. Results of the First Nations Quality Assessment Tool**

|  | 1 | 2 | 3 | 4 | 5 | 6 | 7 | 8 | 9 | 10 | 11 | 12 | 13 | 14 | Total |  |
| --- | --- | --- | --- | --- | --- | --- | --- | --- | --- | --- | --- | --- | --- | --- | --- | --- |
| **1. Cairns et al. 2022 (QLD)** | Y | Y | Un | P | Y | Un | Un | P | Y | Y | Un | Y | Y | Y | 9 | H |
| **2. Carol et al. 2012 (WA)** | Y | Y | Y | Y | Y | Y | Un | Un | Y | Y | Y | Y | Y | Y | 12 | H |
| **3. Dawson et al. 2021 (South Africa)** | Y | Y | Y | Y | Y | Un | Un | Y | Y | Y | Y | Y | Y | Y | 12 | H |
| **4. Du Toit et al. 2014 (South Africa)** | Y | Y | N | N | Y | Un | Un | N | P | Y | Y | Y | P | Un | 7 | M |
| **5. Gidgup et al. 2022 (WA)** | Y | Y | Y | Y | Y | Un | Un | Y | Y | Y | Y | Y | Y | Y | 12 | H |
| **6. Harding et al. 2022 (NZ)** | Y | Y | Y | Y | Y | Un | Un | Un | Y | Y | N | Y | Un | Un | 8 | M |
| **7. Hikaka et al. 2021 a (NZ)** | P | Un | P | Un | Y | Y | Y | Y | Un | Un | Y | Y | Y | Un | 8 | M |
| **8. Hikaka et al. 2021 b (NZ)** | Y | Y | Y | Y | Y | Y | Y | Y | Y | Un | Un | Y | Y | Y | 12 | H |
| **9. Lavrencic et al. 2021 (NSW)** | Y | Y | Y | Y | Y | Un | Un | Un | Y | Y | Y | Y | Un | P | 7.5 | M |
| **10. Mackell et al. 2022 (WA, NT, QLD)** | Un | P | Y | Y | P | Un | Un | Un | N | Y | N | Y | Y | Y | 7 | M |
| **11. Macnivnen et al. 2021 (NSW, WA, SA)** | Y | Y | Un | Un | Un | Un | Un | P | Y | N | N | Y | Y | Y | 6.5 | M |
| **12. Murphy et al. 2012 (WA)** | P | Y | Y | Y | Y | Un | Un | Un | Un | Y | Y | Y | Y | Un | 7.5 | M |
| **13. Oetzel et al. 2020 (NZ)** | Y | Y | Y | Y | Y | Y | Y | Y | Y | Y | Un | Y | Y | Y | 13 | H |
| **14. Pelcastre et al. 2016 (Mexico)** | Un | P | Un | Un | Y | Y | N | P | Y | Y | Y | Un | N | Un | 6 | M |
| **15. Smith, Grundy & Nelson 2010 (NT)** | Y | Y | Y | Y | Y | Y | Un | Y | Y | Y | Y | Y | Y | Y | 13 | H |
| **16. Wettasinghe et al. 2020 (NSW)** | Un | Un | Un | Un | Un | Un | Un | Un | No | Un | Y | N | N | N | 1 | L |

The ATSIQAT consists of 14 questions, constituting 4 possible responses. Considering responses outlined by the ATSIQAT – being yes, partially, no, or unclear, it was determined that the number of yes affirmations totaling 9 and upwards out of 4 questions, would constitute a high degree of alignment (with two partial concessions totaling one yes), 5 – 8 yeses would constitute a medium grading, while 1 – 4 yeses would represent a low grading.

### **Supplementary Table 5. Results of the JBI Quality Assessment Tool**

|  | **1** | **2** | **3** | **4** | **5** | **6** | **7** | **8** | **9** | **10** | **Whole service**  **vs**  **Single program** | **Total** |  |
| --- | --- | --- | --- | --- | --- | --- | --- | --- | --- | --- | --- | --- | --- |
| **1. Cairns et al. 2022 (QLD)** | Y | Y | Y | Y | Y | Un | Y | Y | n/a | Y | W | 8 | H |
| **2. Carol et al. 2012 (WA)** | Y | Y | Y | Y | Y | N | Y | Y | Y | Y | W | 9 | H |
| **3. Dawson et al. 2021 (South Africa)** | Y | Y | Y | Y | Y | Un | Y | Y | Y | Y | W | 9 | H |
| **4. Du Toit et al. 2014 (South Africa)** | Y | Y | Y | y | N | Y | Y | Y | Un | Y | W | 8 | H |
| **5. Gidgup et al. 2022 (WA)** | Y | Y | Y | Y | Y | Y | Y | Y | Y | Y | S | 10 | H |
| **6. Harding et al. 2022 (NZ)** | Y | Y | Y | Y | Y | N | Y | Y | Y | Y | W | 9 | H |
| **7. Hikaka et al. 2021 a (NZ)** | Y | Y | Y | Y | Y | Un | Y | n/a | n/a | Y | S | 7 | M |
| **8. Hikaka et al. 2021 b (NZ)** | Y | Y | Y | Y | Y | Un | Y | Y | Y | Y | S | 9 | H |
| **9. Lavrencic et al. 2021 (NSW)** | Y | Y | Y | Y | Y | Un | Un | Y | Y | Y | S | 8 | H |
| **10. Mackell et al. 2022 (WA, NT, QLD)** | Y | Y | Y | Y | Y | Un | Y | Y | Y | Y | S | 9 | H |
| **11. Macnivnen et al. 2021 (NSW, WA, SA)** | Y | Y | Y | Y | Y | Un | Un | Y | Y | Un | S | 7 | M |
| **12. Murphy et al. 2012 (WA)** | Y | Y | Y | Y | Y | N | N | N | Un | Y | W | 6 | M |
| **13. Oetzel et al. 2020 (NZ)** | Y | Y | Y | Y | Y | Un | Y | Y | Y | Y | Y | 9 | H |
| **14. Pelcastre et al. 2016 (Mexico)** | Y | Y | Y | Y | Y | No | No | Y | Y | Y | W | 8 | H |
| **15. Smith, Grundy & Nelson 2010 (NT)** | Y | Y | Y | Y | Y | Y | Y | N | Un | Y | W | 8 | H |
| **16. Wettasinghe et al. 2020 (NSW)** | Un | Un | Un | Y | Un | Un | Un | Y | Y | Y | W | 4 | M |

The JBI Critical Appraisal tool consists of 10 questions, constituting 4 possible responses. Considering responses outlined by the JBI Critical Appraisal Tool – being yes, non, unclear, or not applicable, it was determined that the number of yes affirmations totaling 8 and upwards out of the 10 questions would constitute a high degree of alignment, 5 – 7 yeses would constitute a medium grading, while 1 – 4 yeses would represent a low grading.

### **Supplementary Figure 1 – The Family Model of Care**


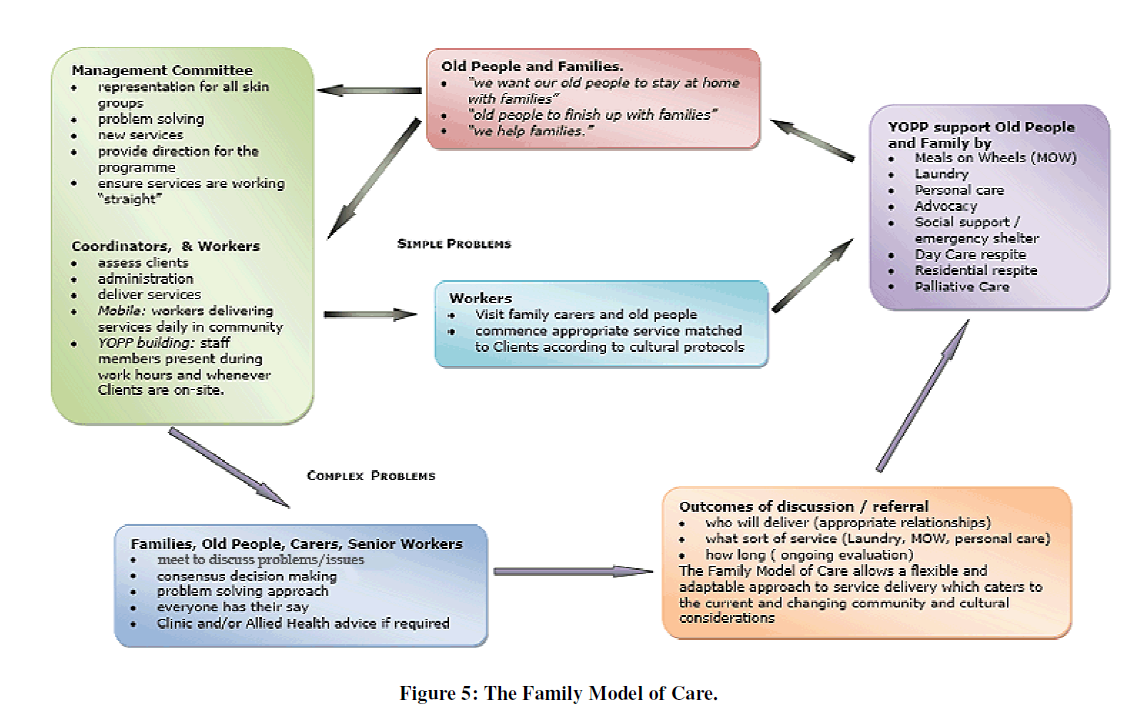


Source: Smith et al. (45)

### **Supplementary Figure 2 – Lungurra Ngoora Remote Community Model of Care**


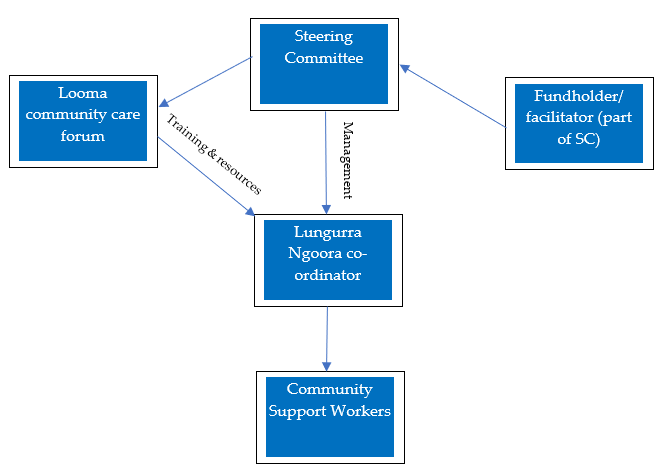


Source: Carroll et al. (41)

**Supplementary Figure 3 – Model for a holistic, healthy ageing program**


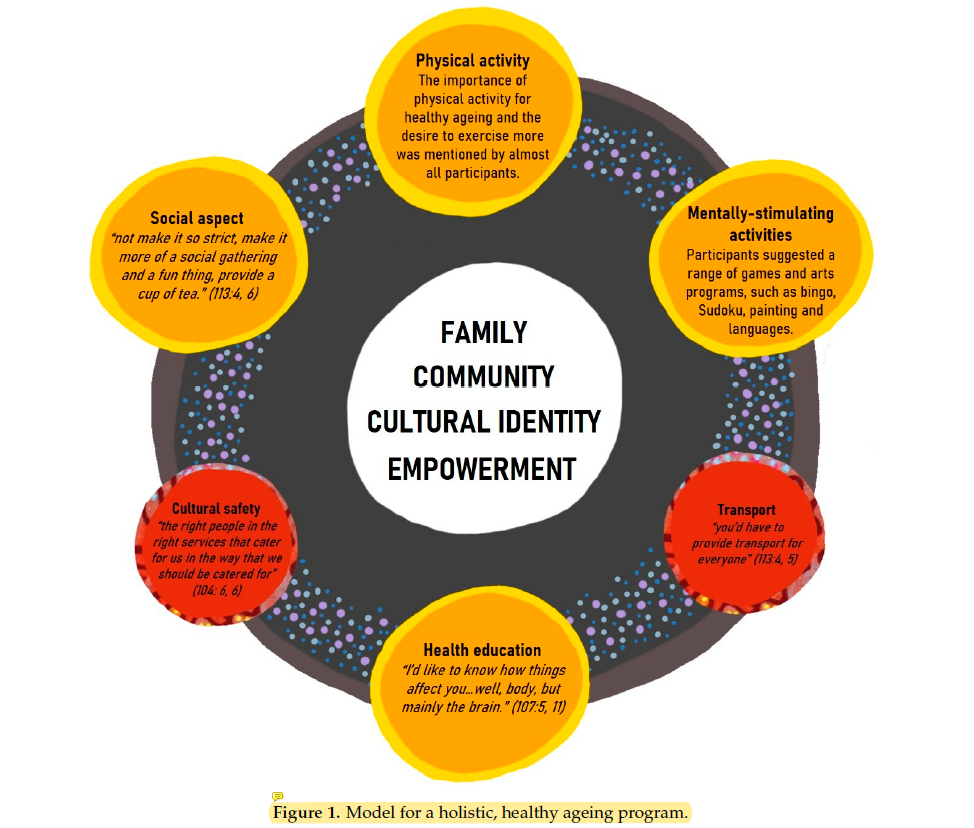


Source: Wettasinghe et al. (46)

**Supplementary Figure 4 – Good Spirit, Good Life Assessment Domains**


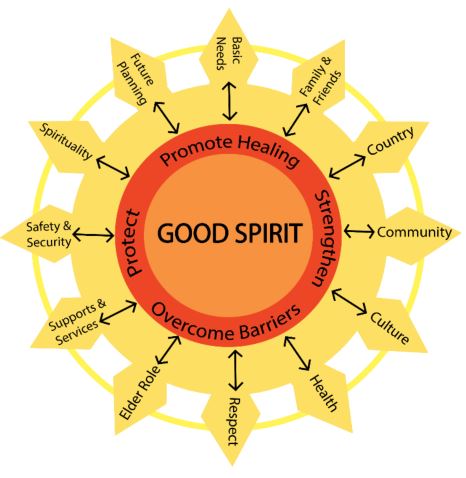


Source: Smith et al. (12)
